# Food label granularity and working memory: effects on food choice in a randomized controlled trial

**DOI:** 10.1186/s41043-025-01076-x

**Published:** 2025-10-24

**Authors:** Constanza Avalos

**Affiliations:** Department of Social Statistics, University of Manchester, Manchester, UK

**Keywords:** Food labeling, Memory short-term, Choice behavior, Food preferences, Randomized controlled trial

## Abstract

While prior research on front-of-package labels has focused on their design, the impact of their informational **granularity**—or level of detail—on consumer choice remains understudied. This study aimed to investigate how the informational granularity of food calorie labels, in conjunction with consumers’ working-memory capacity, influences choice behavior. Methods: A randomized controlled trial was conducted (UK adults, *N* = 498) in which participants completed a cereal-shopping task after being assigned to one of three conditions: coarse label, detailed label, or no label. Working-memory capacity was evaluated via a three-level n-back test. The primary outcomes measured were the average number of calories per chosen product and the probability of selecting low-calorie options. Multilevel models were employed to test both main and interaction effects, with the Benjamini–Hochberg adjustment applied to control the false discovery rate. Results: Compared with the control condition, food labels reduced mean calorie selection by 4.56%. Each one-unit increase in 3-back performance resulted in an additional 14 kcal reduction under coarse labels and 18 kcal under detailed labels. Individuals with high working memory capacity exhibited a preference for moderate-calorie products over the lowest-calorie products when detailed labels were displayed. The effects of capacity under coarse labels were less pronounced and primarily limited to avoiding the lowest-calorie option. These findings provide conceptual insights into information optimization and offer practical guidance for policymakers on designing effective FOP schemes by specifying when detail helps versus hinders consumer choice.

**Supplementary Information:**

The online version contains supplementary material available at 10.1186/s41043-025-01076-x.

## Introduction

High-energy-dense foods have been identified as critical factors driving the increase in obesity rates, thereby increasing the population’s risk of developing noncommunicable diseases such as type 2 diabetes, cardiovascular disease, and various forms of cancer [1, 2]. This trend has led governments worldwide to explore strategies to promote healthier eating choices, with front-of-package labelling (FOP) emerging as a key public health intervention. By providing concise and simplified ‘at-a-glance’ assessments of the nutritional content of foods, these labels aim to promote dietary quality in two ways: (1) improving the understanding of the nutritional quality of packaged foods [3–5] and (2) driving product reformulation [6, 7].

Traditionally, the nutritional facts displayed on packaging have been the primary source of data that many nations have relied upon to guide consumers toward making healthier food choices [8]. However, nutritional facts are challenging to comprehend, and there is limited evidence to suggest that this approach effectively influences dietary behaviors in a positive manner [9, 10]. Given that FOP labels have been demonstrated to be more salient and easier to comprehend, there has been a growing emphasis on optimizing the use of FOP labels to enhance dietary quality [3, 4, 11, 12]. Recent systematic reviews incorporating experimental and observational data have shown that labelling can decrease consumer energy intake by 6.6% and increase vegetable consumption by 13.5%, helping individuals identify healthier food options [4], although the impact on purchasing intentions remains ambiguous, and the effects on overall consumption are limited [3].

From a standard economic perspective, FOP labelling can be viewed as a disclosure policy that aims to address information asymmetry [13]. Labelling introduces transparency [14] and respects consumer freedom of choice [15] without imposing stringent regulations. Research indicates that individuals tend to exhibit preference biases and misjudge the health consequences of their behaviors [13, 16]. Consequently, implementing FOP labelling is a justified way to educate individuals about potential costs that may not be fully internalized at the time of purchase [2]. By making information available, labelling aims to encourage more informed decision-making, helping consumers better align choices with long-term health interests. More specifically, FOP labels can influence the mechanisms underlying belief and behavioral intention, as well as those associated with planning, goal setting, and the maintenance of the behavior of interest, namely, the use of FOP labels during the purchase of high-energy-dense foods [17, 18]. However, the expected effects of this policy are ambiguous, as they depend on the prior beliefs of people about the healthiness of their shopping baskets, which may be updated after the use of labelling [12, 14]. Even if individuals have perfect knowledge of the nutritional content of products, FOP labelling can still influence their choices by prompting a heuristic learning process for food decisions [4, 11].

People’s understanding of labelling is central to the efficacy of this policy. As the Grunert and Mills theoretical framework suggests, for a label to be effective, it should be seen, liked, and understood [19]. The literature indicates that labels have been found to enhance individuals’ ability to select products on the basis of health status when they can objectively understand the information they contain, enabling them to accurately differentiate products according to their nutritional content [20–25]. Additionally, labels have been shown to improve product selection abilities when individuals subjectively perceive that they have understood the nutritional information presented [20–22, 26]. Demographic attributes, personal interest, health literacy levels, and label design have all been identified as factors that can influence the understanding of labels [19, 27].

**Fig. 1:**
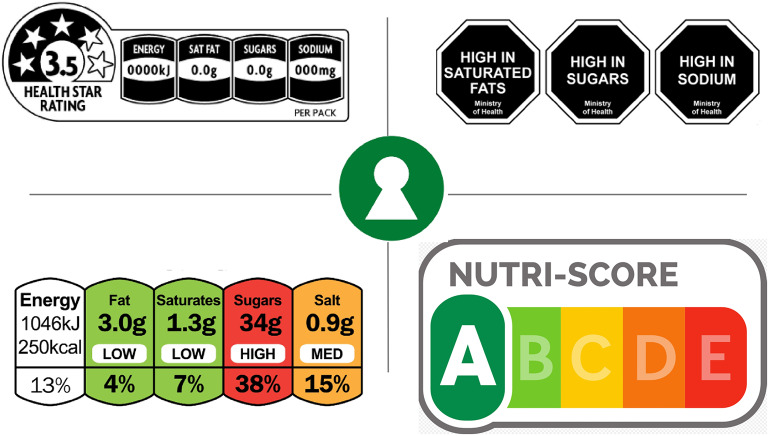
FOP label granularity. At the top are Healthy Start Rating (4 chunks) and Warning Labels (1 chunk). In botton, Multiple Traffic Light (4 chunks) and Nutri-Score (5 chunks) were used. In the middle, KH (1 level)

In psychology, the way in which labelled information is provided is crucial [7, 13]. As individuals exhibit limited attention and cognitive resources, they face a trade-off between the time and effort required to locate and interpret information [28–30]. Therefore, a key factor is the simplicity of the information used [16]. The literature has not yet reached a definitive consensus on which FOP labelling format is the most readily legible and understandable for consumers and which consequently promotes the healthiest purchasing decisions. Existing evidence suggests that interpretative labels, which convey information about the healthiness of a food, tend to be more effective than noninterpretive systems that do not involve any judgement, such as reference intakes. Prominent examples of interpretative labels include nutrient-specific systems such as Multiple Traffic Light (MTL) and Healthy Start Rating (HSR); nutrient-specific warnings such as Keyhole (KH) and Warning Labels (WL); and summary indicators such as the Nutri-Score (NS) (see Fig. 1). Nutrient-specific systems and summary labels integrate numerical information and visually accessible elements, in contrast with warning labels, which succinctly convey a product’s overall health without delving into comprehensive details [11, 31, 32].

FOP labels are designed to condense complex nutrient declarations into intuitive summaries that help consumers identify healthier products at a glance. While prior work has examined colour, shape, and evaluative framing, far less attention has been given to granularity—the degree of detail or the number of informational “chunks” contained in a label [3–5]. Conceptually, granularity captures how finely a scheme partitions the healthfulness continuum; for example, Nutri-Score and Multiple Traffic Lights categorize products into five ordered levels, whereas warning symbols typically offer only one or two evaluative cues. Early evidence indicates that more finely grained schemes outperform binary warnings in nudging choices toward healthier options because additional strata convey nuance that aids nutritional understanding [11]. However, the cognitive cost of such detail has rarely been considered [33–36].

Cognitive-load theory posits that working memory can handle only a limited number of chunks at any moment; exceeding this capacity impairs comprehension and recall [37]. Classical estimates place this limit at approximately seven items [38], although conventional resource models emphasize flexible allocation rather than a fixed span [39]. Consequently, a label that is optimal for a high-capacity consumer may overwhelm a shopper with lower capacity. Despite this theoretical importance, the literature offers little guidance on exactly how many chunks maximize usability across heterogeneous audiences, leaving regulators to choose granularity largely on pragmatic or political grounds.

The present study addresses this gap by testing whether the effectiveness of FOP labels depends on, and can be optimized for, consumers’ working-memory capacity. Granularity was manipulated via two calorie labels, a coarse, four-chunk format and a detailed, eight-chunk format, which were chosen to bracket the five-chunk MTL standard familiar to UK shoppers. The participants’ working memory was indexed with a three-stage n-back test, permitting a direct examination of the label and cognitive-capacity interaction. Formally, we hypothesized a moderation effect whereby the direction and magnitude of the granularity impact would vary as a positive function of n-back performance. We anticipate a cognitive stratification pattern in which coarse labels promote healthier choices among individuals with lower working memory due to their reduced cognitive demands. Conversely, detailed labels should benefit those with higher capacity by offering more comprehensive diagnostic information. By integrating granularity with cognitive-load theory, our study contributes both conceptually and practically. Conceptually, it reframes label design as a problem of information-chunk optimization under resource constraints. Practically, the findings can inform the European Union’s ongoing deliberations on a harmonized FOP scheme by specifying when additional detail helps and when it hinders consumer decision-making.

## Methods

### Study design

We conducted a randomized controlled trial to investigate the influence of FOP labels on individual breakfast cereal choices in an online choice setting. The participants were randomly assigned to one of three groups: a coarse labelling group, in which cereals displayed less granular labelling; a detailed labelling group, in which products conveyed more granular labelling; and a control group, in which products did not display labels. The allocation was concealed from the participants. Breakfast cereals were selected for this research because of their widespread consumption and the established variability in their nutritional content [14]. The online experimental design allowed for the controlled presentation of nutritional information, mimicking a real-world grocery shopping scenario.

Prolific Limited (Ltd.) recruited participants, providing a national representative probabilistic sample in Great Britain. As an incentive, the subjects received a £4.5 upon completion. They completed the choice experiment on computer devices; smartphones and tablets were not allowed. The experiment was designed on the Qualtrics online platform via several randomization features.

### Outcome measures

The primary outcomes are the average calorie counts of selected products and the probability of selecting lower-calorie products. These measures directly reflect the objective understanding and impact of FOP labels on consumer choices. Additionally, subjective understanding of labelling, assessed through a post experiment questionnaire, is a secondary outcome.

### FOP label granularity

**Fig. 2:**
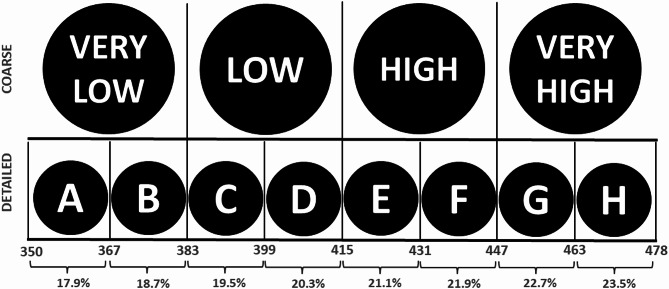
FOP labelling granularity in the choice task, presenting calories per 100 g and the percentage of average daily reference intake (%RI). The “Coarse” condition uses four categories (very low, low, high, very high), while the “Detailed” condition uses an eight-category letter scale (A–H), with each letter corresponding to a specific calorie range (e.g., A: 350–366 kcal; B: 367–382 kcal)

We designed a coarse and detailed label mirroring the structure employed in prior research [40, 41] while adapting the caloric ranges to reflect the breakfast cereals available in this choice experiment (see Fig. 2). For example, A represented approximately 358 kcal, and H represented approximately 471 kcal. To contextualize the calorie information, participants received instructions explaining that a 100 g serving of cereal labelled high would provide, on average, 22% of the recommended daily caloric intake on the basis of a 2,000 kcal diet, whereas a serving labelled A would provide 17.9% of the recommended daily caloric intake. Both labels featured a black circular shape with white text and designs created with Adobe Photoshop software.

### Procedures

We used a similar methodology to that used in previous studies [22, 27, 41]. The study utilized a choice experiment comprising 16 trials, where participants selected breakfast cereals from an online grocery shopping setting. Table 1 presents the eight products included in the experiment, which span a caloric range of 360–470 kcal per 100 g, with the nutritional content verified via manufacturer websites. In this study, low-calorie products are defined as those containing 383 kcal or fewer per 100 g on the basis of United Kingdom (UK) regulations [42]. We established this threshold by taking into account the typical serving sizes for breakfast cereals and with the aim of encompassing products at the lower end of the calorie range within the sample. Cereal brands 1 and 2 from Table 1 met this criterion and were accordingly classified as lower-calorie alternatives. We employed a restricted factorial design to present participants with sets of four cereal brands in each trial.

**Table 1:** Calorie count for breakfast cereals included in the choice experiment (*n* = 8)

Cereal brand	Calorie count (kcal per 100 g)	Coarse	Detailed
None option	0	-	-
1	360	Very low	A
2	374	Very low	B
3	392	Low	C
4	398	Low	D
5	423	High	E
6	431	High	F
7	453	Very high	G
8	470	Very high	H

**Fig. 3:**
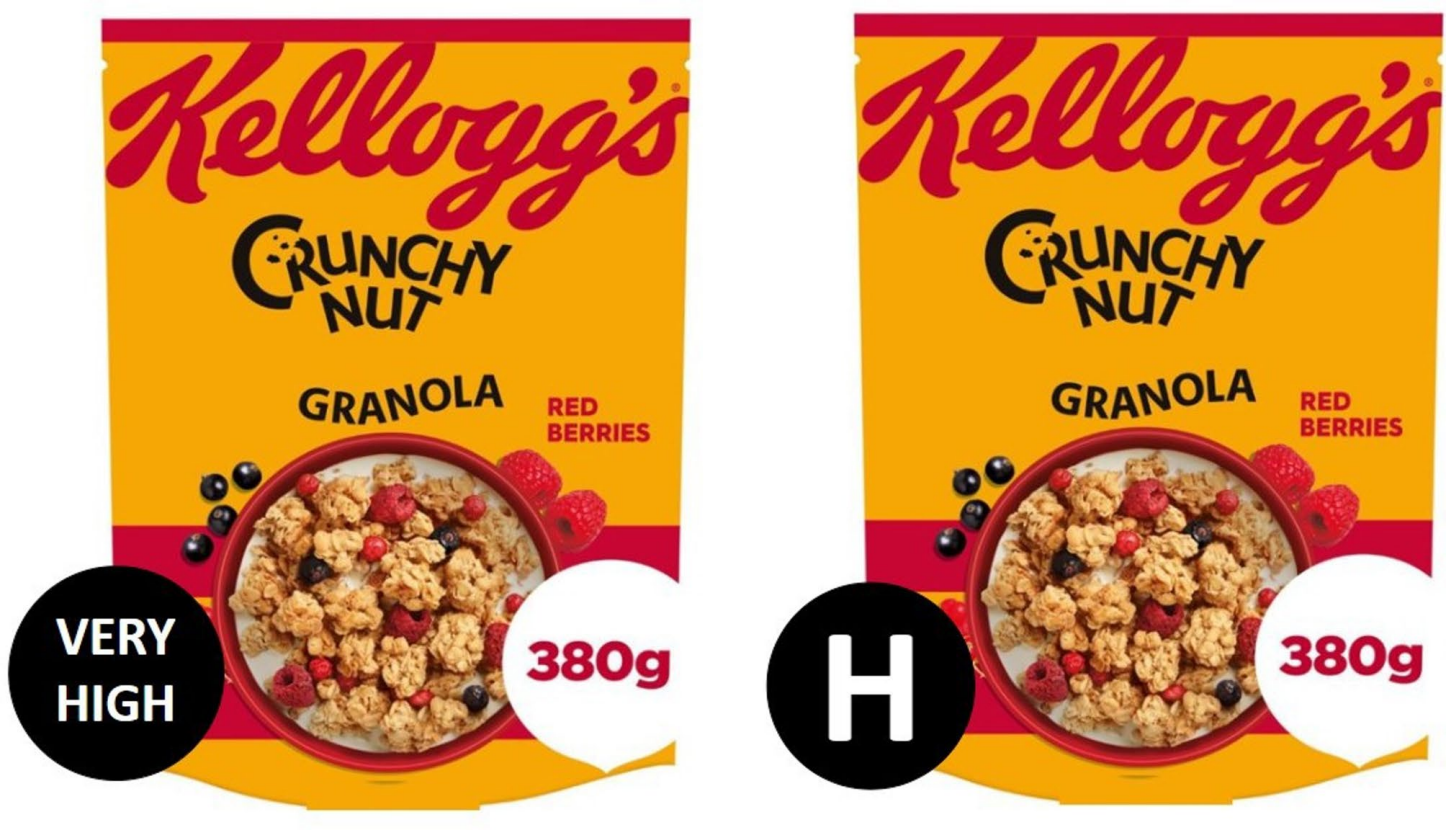
Manipulation of the product front package. A calorie label has been added to the original image

The study mitigated the potential confounding effect of product familiarity by employing authentic product images captured by the researchers and eliciting participants’ self-reported familiarity with the items (see Fig. 3). This approach aims to increase the ecological validity of the online shopping environment [43].

### N-back test

We assessed working memory capacity via a computerized n-back test [44–47]. The participants were presented with a sequence of letters (A, B, C, D, F, H, K, L, M, N, O, P, Q, R, X) displayed individually in the center of the screen with a font size of 30 pt for 1 s for each interval between stimuli. The test utilized a JavaScript program to generate the randomized presentation order of the stimuli comprising three blocks: 1-back, 2-back, and 3-back. The 1-back block is the simplest level, where the stimulus must be compared to the stimulus that immediately preceded it. Examples were provided to the participants prior to commencing the test.

The scoring was based on the performance of the participant, specifically concerning correct identifications (hits) when the stimulus was not presented and when the participant responded positively (false alarms). Each block consisted of 30 trials, with a target of 30 hits each, and did not provide any feedback to participants but provided some examples. The performance was assessed via the d-prime (*d’*) measure, which was calculated as *d’* = *Z*(*hits*) *− Z*(*falsealarms*), where Z represents the inverse of the cumulative normal distribution function applied to the hit rate and false alarm rate, respectively [44]. All the participants began with the 1-back block. To progress to the following blocks, they were required to make fewer than 25 errors in the preceding n-back level. Errors were defined as the sum of false alarms and missed responses to target stimuli (miss). This approach helps mitigate the potential confounding effects of boredom or disengagement on performance in the more demanding stages of the test, a factor particularly relevant in online research environments [48].

### Subjective Understanding and behavioral questionnaire

We included a brief questionnaire to assess participants’ subjective understanding of the FOP labels presented in the choice experiment. The participants reported whether they had sufficient information to make informed choices and whether they recalled seeing the labelling. This approach is similar to that used by [27], where the perceptions of the labels were also assessed. Furthermore, the participants completed a behavioral questionnaire to evaluate their food-shopping behaviors, dietary knowledge, and demographic characteristics.

### Statistical analysis

This study used multilevel logistic regression to evaluate the probability that participants would choose lower-calorie options and multilevel linear regression to estimate the calorie counts among the experimental groups. The model specification is as follows:

$$\begin{gathered}{\text{log}}\left( {\frac{{{p_{ij}}\:}}{{1 - \:{p_{ij}}}}} \right) = {\beta _0} + {\beta _1}FO{P_{ij}} \\ + {\beta _2}\left( {FO{P_{ij}}*n - bac{k_{ij}}} \right) \\ + {\beta _3}Control{s_{ij}} + {u_j} \sim N{\mkern 1mu} (0,{\mkern 1mu} \sigma _u^2) \\ \end{gathered}$$

with *p*_*ij*_ being the probability that the *i* trial within the *j* participant results in the choice of a lower-calorie product. where *β*_0_ represents the intercept (logarithmic odds of the outcome when all predictors equal zero); $\beta_{1}$ captures the effect of different FOP; $\beta_{2}$ estimates the interaction effect between FOP and the n-back test; and $\beta_{3}$ represents control variables that include the choice sets (Trials) and cereal preferences. The term ${\rm{uj }} \sim {\rm{ N (0, }}\theta {\rm{2)}}$ N$N$ (0,$\sigma^{2}$) denotes the participant- specific random intercept, which accounts for the correlation structure induced by the 16 repeated choice trials nested within each individual. This random effect captures unobserved heterogeneity between participants and controls for the nonindependence of observations, thereby providing more accurate standard errors for the fixed effects. The variance parameter o2 quantifies the variability between participants in the baseline propensity to choose lower-calorie options after accounting for the fixed effects in the model. Similarly, multilevel linear regression models were used to compare the calorie counts of the selected products under experimental conditions. The outcome variable $Y_{ij}$ in these models represented the average calorie count of the products chosen by participant j in trial i, including an error term ej. In all regressions, standard errors were clustered at the participant level.

The data collection process excluded participants if they self-reported not buying or eating cereal in the past twelve months to ensure that the lack of familiarity with the products did not affect the findings. The analyses were not blinded but were carried out by a statistician-in-training who had participated in the preparation and conduct of the experiment. All analyses were performed via R statistical software [49], and we reported a significance level of 5%.

## Results

### Participant characteristics

**Fig. 4:**
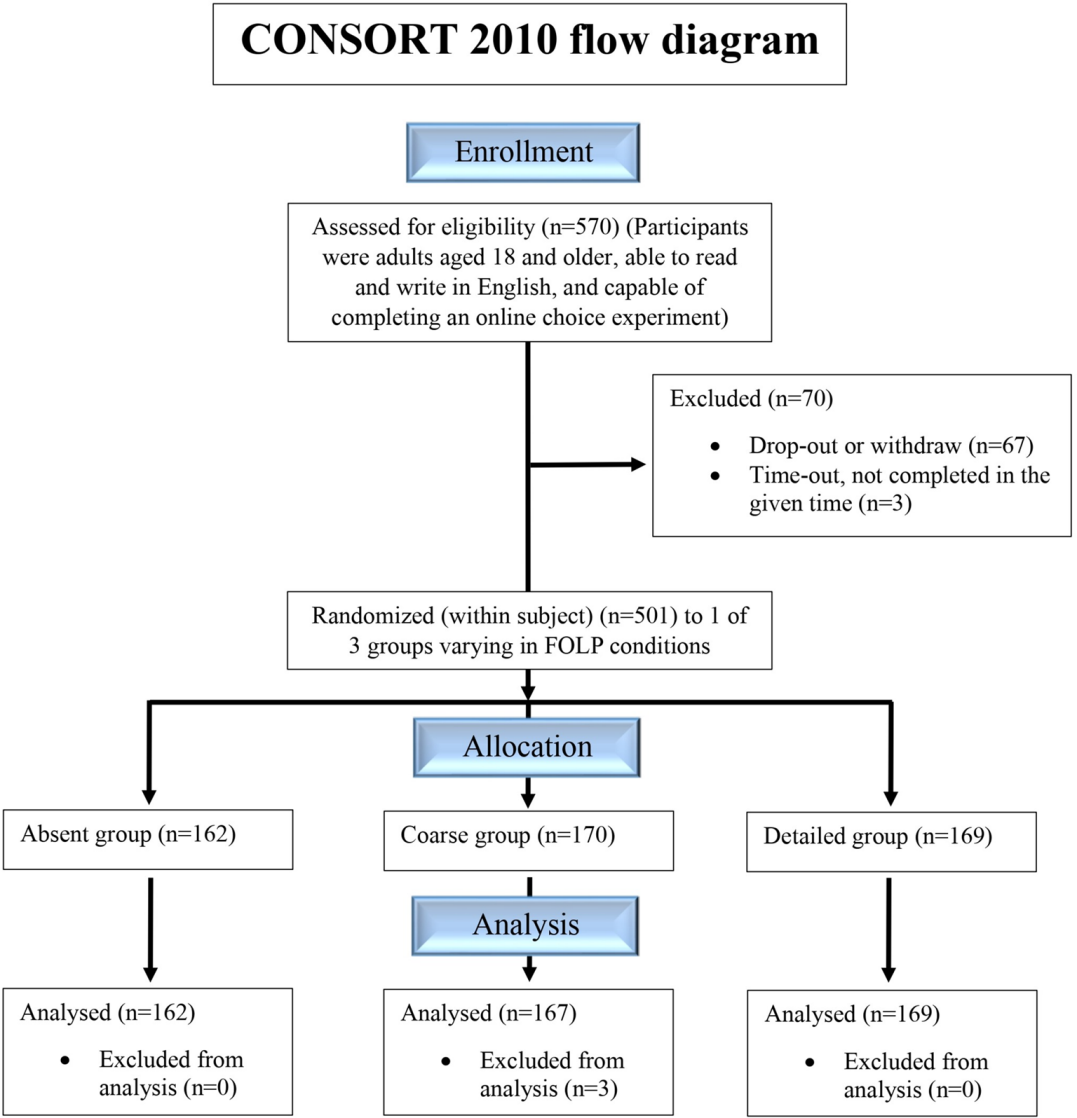
Consort flow diagram reporting recruitment and randomization

The participant flowchart for recruitment and randomization is presented in Fig. 4. Data were collected from 29th August–10th September 2024 from participants aged 18 years and older who were able to complete an online experiment. Prolific Ltd. invited 570 panel members to participate, and the complete data of 498 participants were considered in the analysis. This represents 87.3% of the recruited sample. Of the original participants, 67 withdrew from the study, and data from 3 participants were excluded from the analysis because they exceeded the maximum allotted time for providing responses. Individuals were randomly divided into three different experimental conditions, and the sample sizes were similar across groups: absent (*n* = 162), coarse (*n* = 170) and detailed (*n* = 169). Three participants were excluded from the detailed group after randomization because of incomplete data. Individuals took 22 min to complete the randomized control trial on average, which included the choice task, n-back test, and posterior behavior questionnaires.

Table 2 shows the characteristics of the participants across the experimental groups. No differences in demographic characteristics were observed between the groups (*χ*^2^*p >* 0.05). In total, approximately half of the participants (52%) were female, most identified as White (84%), nearly one-third had a higher education level (40%), and half had £30,001 - Above £40,000 income (54%). Table 3 presents the household composition and food purchasing behaviors of the experimental groups.

**Table 2:** Participant characteristics by experimental group (*n* = 498)

Variable	Alternative	All	Absent *n* (%)	Coarse *n* (%)	Detailed *n* (%)	χ^2^ (*p*)
Participants		498	162 (32)	167 (34)	169 (34)	
Sex	Female	257 (52)	86 (54)	91 (55)	80 (47)	0.39
Sex	Male	240 (48)	75 (46)	75 (45)	89 (53)	
Sex	Unreported	1 (0)	1 (0)			
Age	18–34	140 (28)	44 (27)	46 (28)	50 (29)	0.94
Age	35–54	165 (33)	55 (34)	58 (35)	52 (31)	
Age	55–65+	193 (39)	63 (39)	63 (37)	67 (40)	
Ethnicity	White	417 (84)	138 (85)	139 (83)	140 (83)	0.89
Ethnicity	Mixed	75 (15)	23 (14)	26 (16)	26 (15)	
Ethnicity	Unreported	6 (1)	1 (1)	2 (1)	3 (2)	
Education	Higher	199 (40)	59 (36)	66 (40)	74 (44)	0.57
Education	Lower	297 (60)	102 (63)	101 (60)	94 (56)	
Education	Unreported	2 (0)	1 (1)	0 (0)	1 (1)	
Income^1^	Higher	267 (54)	93 (57)	96 (58)	78 (46)	0.13
Income	Lower	195 (39)	60 (37)	57 (34)	78 (46)	
Income	Unreported	36 (7)	9 (6)	14 (8)	13 (8)	
BMI ^1^	Obesity	130 (26)	45 (28)	38 (23)	47(28)	0.95
BMI	Overweight	159 (32)	52 (32)	55 (33)	52 (31)	
BMI	Normal weight	191 (38)	59 (36)	68 (41)	64(38)	
BMI	Underweight	18 (4)	6 (4)	6 (4)	6 (4)	

^1^BMI (body mass index) was estimated with self-reported height and weight

^2^Higher income: £30,001 - Above £40,000; Lower income: Below £10,000 - £30,000

Approximately one-third of the participants had children in the household (32%), and the majority had food-shopping responsibilities (always = 51%, most of the time = 27%), whereas nearly half indicated that they are currently trying to lose weight (45%). A large proportion of the participants reported that they examined product ingredients while shopping (most of the time = 36%, approximately half of the time = 22%). The subjects reported similar proportions of familiarity across the experimental conditions (very familiar = 35%, somewhat familiar = 55%).

We evaluated participants’ preferences for the cereal brands in the choice experiment by asking them to complete a ranking task, where they ordered the products on a Likert scale (1 = most preferred to 8 = least preferred). Table 4 shows the mean rank and standard deviation (SD) for each cereal brand; lower mean ratings indicate greater preference. Cereal brand 2 (mean: 3.60; SD: 2.60) was the most preferred average, whereas brand 7 (mean: 5.91; SD: 2.33) was the least preferred. The ranking metrics for product preferences were not similar across the experimental groups. The preference ranking for cereal brand 4 differed significantly across the experimental conditions according to an analysis of variance (ANOVA *p <* 0.05). Analyses revealed that participants in the coarse group (mean: 3.75; SD: 1.95) ranked this product lower than those in the absent condition (mean: 4.31; SD: 1.94). Similarly, cereal 6 showed differences in preference rankings across conditions (ANOVA *p <* 0.05). The participants in the coarse condition (mean: 4.26; SD: 1.86) showed a significantly greater preference for cereal 6 than did those in the absent condition (mean: 3.61; SD: 1.88).

**Table 3:** Household composition and purchasing behaviors by experimental group (*n* = 498)

Variable	Alternative	All *n* (%)	Absent *n* (%)	Coarse *n* (%)	Detailed *n* (%)	χ2 (*p*)
		498	162 (32)	167 (34)	169 (34)	
Children at home	No	334 (67)	103 (64)	108 (65)	123 (73)	0.39
Children at home	Yes	157 (32)	56 (35)	57 (34)	44 (26)	
Children at home	Unreported	7 (1)	3 (1)	2 (1)	2 (1)	
Losing weight	No	224 (45)	70 (43)	75 (45)	79 (47)	0.47
Losing weight	Yes	268 (54)	91 (56)	91 (54)	86 (51)	
Losing weight	Unreported	6 (1)	1 (1)	1 (1)	4 (2)	
Responsibility	Always	255 (51)	83 (51)	87 (52)	85 (50)	0.99
Responsibility	Most of the time	135 (27)	45 (28)	45 (27)	45 (27)	
Responsibility	Half of the time	60 (12)	18 (11)	20 (12)	22 (13)	
Responsibility	Sometimes	43 (9)	15 (09)	13 (07)	15 (08)	
Responsibility	Never	5 (1)	1 (1)	2 (1)	2 (1)	
Check ingredients	Always	58 (12)	23 (14)	20 (12)	15 (09)	0.07
Check ingredients	Most of the time	177 (36)	58 (36)	70 (42)	49 (29)	
Check ingredients	Half of the time	111 (22)	31 (19)	36 (22)	44 (26)	
Check ingredients	Sometimes	132 (27)	43 (27)	33 (20)	56 (33)	
Check ingredients	Never	20 (4)	7 (4)	8 (8)	5 (3)	
Familiarity	Very familiar	174 (35)	52 (32)	70 (41)	52 (31)	0.20
Familiarity	Somewhat familiar	272 (55)	90 (56)	82 (50)	100 (60)	
Familiarity	Neither	20 (4)	6 (04)	5 (03)	9 (05)	
Familiarity	Unfamiliar	32 (6)	14 (8)	10 (6)	8 (4)	

For cereal 8, the detailed condition also significantly affected the preference rankings (ANOVA *p <* 0.05). The participants in the coarse (mean: 4.60; SD: 2.07) and detailed conditions (mean: 4.49; SD: 2.11) showed significantly greater preferences than did those in the absent condition (mean: 3.88; SD: 2.13).

### N-back performance across experimental groups

Table 5 presents the distributions of participants’ *d’* scores across the experimental groups for each level of the n-back block. To account for potential boundary issues where *d’* scores might be undefined owing to hit or false-alarm rates of 0 or 1, we applied the correction method described by Stanislaw and Todorov [50]. Specifically, any score of 0 was replaced with 0.5/n, and any score of 1 was replaced with (*n* − 0.5)/*n*, where n represents the number of target and noise trials. In this study, the target was equal to 30, as each block contained 30 trials, and the noise was established at 48, the highest observed false alarm rate across all participants.

The sample sizes for the 1-back, 2-back, and 3-back conditions were 498, 464, and 452, respectively. This represents a reduction of 7.33% and 10.18% from the initial sample size, likely due to participant data exclusion on the basis of predefined criteria. As hypothesized, n-back test performance declined as task difficulty increased. The 3-back condition presented the lowest mean *d’* scores across all treatment groups (absent: 2.29 (SD 0.87), coarse: 2.29 (SD 0.82), and detailed: 2.16 (SD 0.90)). While the mean *d’* scores and standard deviations were relatively consistent across groups, no differences were observed in scores between groups (ANOVA *p >* 0.05). The total duration of the n-back test averaged approximately 11 min (SD = 3.25 min) across all groups.

**Table 4:** Product preferences by experimental group, means and SDs (*n* = 498)

Cereal brand	Calorie brands	Absent *n* (%)	Coarse *n* (%)	Detailed *n* (%)	Anova (*p*)
		162 (0.32)	167 (0.34)	169 (0.34)	
1	360	4.31 (2.37)	4.07 (2.42)	3.98 (2.36)	0.43
2	374	3.49 (2.49)	3.39 (2.56)	3.91 (2.74)	0.15
3	392	4.98 (1.83)	4.55 (2.04)	4.66 (1.93)	0.12
4	398	4.31 (1.94)	3.75 (1.95)	3.93 (2.00)	0.03
5	423	5.47 (2.03)	5.32 (1.97)	5.31 (2.17)	0.74
6	431	3.61 (1.88)	4.26 (1.86)	3.96 (1.79)	0.00
7	453	5.94 (2.37)	6.04 (2.24)	5.76 (2.37)	0.54
8	470	3.88 (2.13)	4.60 (2.07)	4.49 (2.11)	0.00

**Table 5:** *D’* scores by experimental group, *n* (Mean, SD)

Variable	1-back (*n* = 498)	2-back (*n* = 464)	3-back (*n* = 452)
Absent	162 (3.37, 1.01)	157 (3.24, 1.00)	150 (2.29, 0.87)
Coarse	167 (3.13, 1.26)	150 (3.24, 0.91)	148 (2.29, 0.82)
Detailed	169 (3.21, 1.17)	157 (3.14, 0.93)	154 (2.16, 0.90)
Anova (*p*)	0.16	0.59	0.30

### Treatment effect on calorie counts

The results from multilevel linear regression analyses examining the effects of FOP labels on calorie counts are presented in Table 6. All the models were adjusted for choice sets (Trials) and product preferences. The baseline model (Model 1) indicated negative but not significant direct effects of either Coarse (-9.14, standard error [SE] = 7.20) or Detailed labels (-3.46, SE = 7.18) on calorie counts. These effects remained stable after controlling for choice sets (Model 2) and product preferences (Model 3). However, the introduction of n-back performance interactions revealed notable heterogeneous effects. Particularly compelling were the significant negative interactions between labelling and 2-back performance (Model 5): both Coarse*2-back (-14.51, SE = 6.90, *p <* 0.05) and Detailed*2-back (-17.35, SE = 6.75, *p <* 0.05) interactions indicate that as participants’ performance on the n-back test improves (reflected by a unit increase in d’), the average caloric count decreases. This pattern persisted in the 3-back block (Model 6), where detailed labels had a stronger moderating effect (-18.05, SE = 7.42, *p <* 0.05) than coarse labels did (-13.52, SE = 7.83, *p <* 0.1). The 1-back block showed similar but less pronounced interaction patterns.

**Fig. 5:**
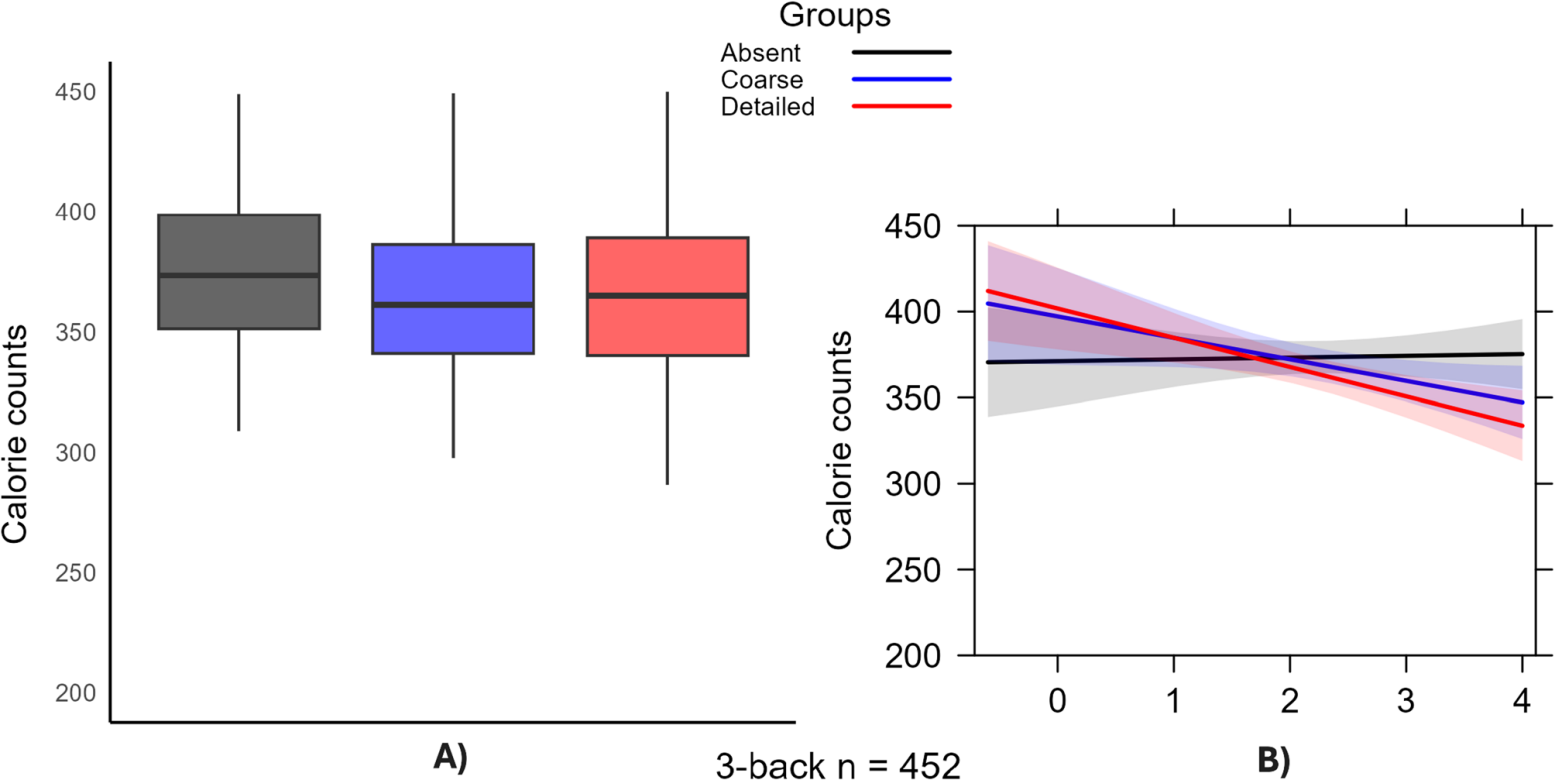
**A**) Effects of FOP labelling on calorie counts by experimental conditions, including 3-back test performance levels as an interaction term. **B**) Plot showing the interaction effects between d’ as 3-back performance and different labelling conditions on caloric count

In Model 6 and Fig. 5A), the Absent group (intercept) had an average calorie count of 361.93 kcal, the Coarse*3-back group averaged 348.41 kcal (3.88% decrease versus Absent), and the Detailed*3-back group averaged 343.88 kcal (5.24% decrease versus Absent). Figure 5B) displays nonparallel trend lines for the different labelling conditions, indicating that the impact of the labelling on the calorie count varies across the *d’* values.

Table 7 presents Hedges-adjusted Cohen’s *d* values alongside model-based means and their 95% confidence intervals. Across the working-memory spectrum *d’*, most Cohen’s *d* values fluctuate between − 0.30 and + 0.30, with nearly every confidence band intersecting 0, indicating that the observed differences are not only quantitatively small but also statistically imprecise. A notable exception is the Detailed-versus-Absent contrast at the highest 3-back level, where the effect reaches *d* = − 0.28 (95% CI [0.50, 0.06]); this approximates the lower threshold of a “small” effect by conventional standards. However, the upper limit remains considerably below the threshold for a moderate effect, suggesting only a modest practical benefit.

**Table 6:**
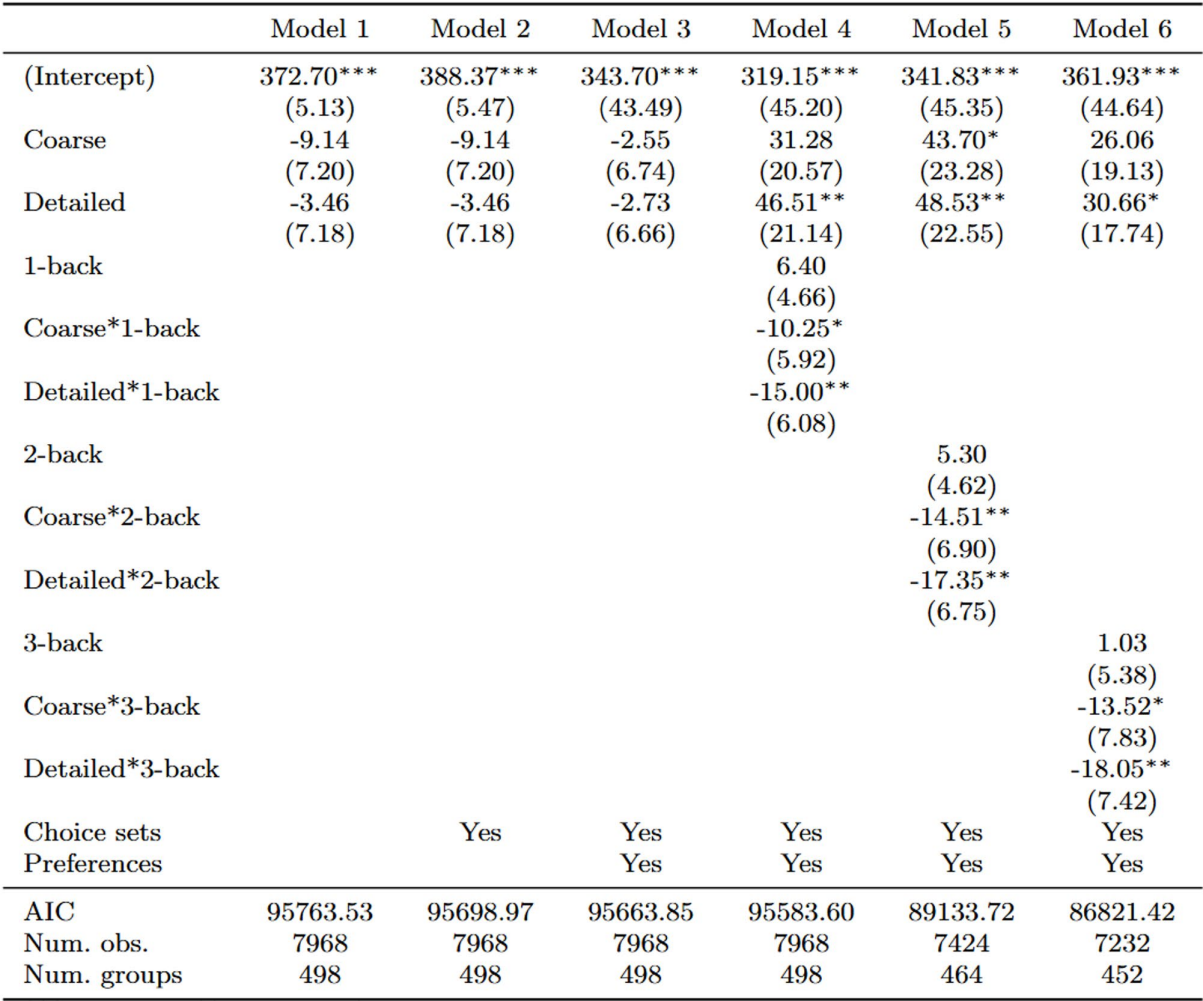
Multilevel linear regression results: effects of FOP labelling on calorie count by experimental conditions, including n-back test performance levels as an interaction term (adjusted for product preferences and choice sets (Trials)

***p < 0.01; **p < 0.05; *p < 0.1

### Treatment effect on the probability of choosing lower-calorie products

Table 6 presents the multilevel log-binomial regression results, including interactions between the labelling groups and the 3-back block adjusted for controls. The analysis spans the full range of available calorie options, from the lowest-calorie (360 and 374 kcal) to the highest-calorie products (453 and 470 kcal). For the lowest-calorie options, contrasting patterns emerge. At 360 kcal, the Coarse*3-back interaction is negative and significant (-0.80, SE = 0.36, *p <* 0.05), indicating that participants with higher working memory capacity, reflected by a unit increase in *d’*, were less likely to select this low-calorie option when presented with coarse labels. The Detailed*3-back interaction has no significant effect (0.11, SE = 0.33).

In the moderate-calorie range (392 kcal), both interaction terms are positive and significant (Coarse*3-back 0.69, SE = 0.41, *p <* 0.1; Detailed*3-back 0.90, SE = 0.39, *p <* 0.05), suggesting that participants with higher d’ values were more likely to select this moderate-calorie option when presented with either type of labelling. For the 398- calorie option, neither interaction term reached statistical significance (Coarse*3-back 0.01, SE = 0.61; Detailed*3-back 0.06, SE = 0.58). For higher-calorie options (423–470 kcal), none of the interaction terms reached statistical significance, although there was a trend toward negative coefficients for Detailed*3-back interactions, particularly for the 423-calorie (-0.48, SE = 0.32) and 431-calorie (-0.51, SE = 0.38) options.

**Table 7:** Predicted cereal–selection position and pairwise cohen’s *d* by d’ level

Index	d’	Group	Mean (95% CI)	Cohen’s d	(95% CI)
				vs. Absent	vs. Coarse
2-back	2.26	Absent	367.2 (354.6, 379.8)	–	+ 0.12 (–0.10, 0.34)
		Coarse	378.1 (364.5, 391.7)	–0.12 (–0.34, 0.10)	–
		Detailed	376.5 (364.0, 389.0)	–0.10 (–0.32, 0.12)	+ 0.02 (–0.20, 0.24)
2-back	3.21	Absent	372.3 (363.2, 381.3)	–	–0.03 (–0.25, 0.19)
		Coarse	369.4 (360.1, 378.6)	+ 0.03 (–0.19, 0.25)	–
		Detailed	365.1 (356.1, 374.1)	+ 0.08 (–0.14, 0.30)	+ 0.05 (–0.17, 0.27)
2-back	4.15	Absent	377.2 (364.9, 389.6)	–	–0.18 (–0.40, 0.04)
		Coarse	360.7 (347.7, 373.7)	+ 0.18 (–0.04, 0.40)	–
		Detailed	353.8 (340.5, 367.0)	+ 0.25 (0.03, 0.47)	+ 0.07 (–0.15, 0.29)
3-back	1.38	Absent	372.5 (359.4, 385.7)	–	–0.08 (–0.30, 0.14)
		Coarse	379.9 (366.1, 393.8)	+ 0.08 (–0.14, 0.30)	–
		Detailed	378.3 (366.4, 390.2)	+ 0.06 (–0.16, 0.28)	–0.02 (–0.24, 0.20)
3-back	2.25	Absent	373.4 (364.3, 382.6)	–	+ 0.05 (–0.17, 0.27)
		Coarse	369.1 (359.9, 378.3)	–0.05 (–0.27, 0.17)	–
		Detailed	363.5 (354.5, 372.5)	–0.11 (–0.33, 0.11)	–0.06 (–0.28, 0.16)
3-back	3.11	Absent	374.3 (361.6, 387.0)	–	+ 0.17 (0.05, 0.39)
		Coarse	358.3 (345.4, 371.3)	–0.17 (0.39, 0.05)	–
		Detailed	348.8 (335.8, 361.9)	–0.28 (0.50, 0.06)	–0.11 (0.33, 0.11)

Across calorie ranges, only two interaction effects showed practical relevance (see Table 9). At 360 kcal, the Coarse*3-back interaction had a small-to-medium effect size (*d* = − 0.44, 95% CI [–0.83, − 0.05]). Conversely, at 392 kcal, the Detailed*3-back interaction produced the largest positive effect (*d* = 0.49, 95% CI [0.08, 0.92]). For all other calorie levels, the Cohen’s *d* confidence intervals included zero, denoting uncertain moderation by working memory. Overall, label granularity alters choice behavior only at specific calorie points, with coarse labels dampening and detailed labels enhancing healthier selections, but these effects are small and context dependent.

See Appendix B for the results of the 2-back and 1-back blocks, which showed similar directional patterns to the 3-back findings for lower-calorie products (360 and 374 kcal), but with notably weaker effects. This suggests that the influence of FOP labels on lower-calorie product selection was most pronounced under higher cognitive load conditions.

**Table 8:**
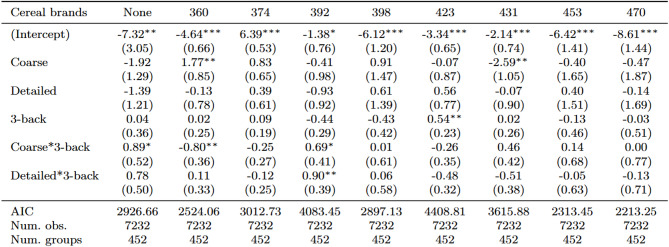
Multilevel log-binomial regression results—effects of FOP labelling on the probability of choosing lower-calorie cereal brands by experimental groups with the 3-back level as the interaction term (adjusted for product preferences and choice sets (Trials)

***p < 0.01; **p < 0.05; *p < 0.1

**Table 9:** Cohen’s *d* (± 95% CI) for the 3-back interaction terms

Cereal brands	Coarse*3-back	Detailed*3-back
None	0.49 [*−* 0.07, 1.05]	0.43 [*−* 0.11, 0.97]
360	*−*0.44 [*−* 0.83, *−* 0.05]	0.06 [*−* 0.30, 0.42]
374	*−*0.14 [*−* 0.43, 0.15]	*−*0.07 [*−* 0.34, 0.20]
392	0.38 [*−* 0.06, 0.82]	0.49 [0.08, 0.92]
398	0.01 [*−* 0.65, 0.67]	0.03 [*−* 0.59, 0.66]
423	*−*0.15 [*−* 0.52, 0.24]	*−*0.27 [*−* 0.61, 0.08]
431	0.25 [*−* 0.20, 0.71]	*−*0.28 [*−* 0.69, 0.13]
453	0.08 [*−* 0.66, 0.81]	*−*0.03 [*−* 0.71, 0.65]
470	0.00 [*−* 0.83, 0.83]	*−*0.07 [*−* 0.84, 0.70]

### Subjective understanding

Table 10 shows the results of the subjective FOP understanding questions by experimental groups. The participants in the coarse labelling condition consistently demonstrated the highest FOP recall rate, reaching 48.0%, whereas those in the detailed labelling condition reported a rate of 40.0%. The perceptions of information sufficiency differed significantly across the experimental groups (*χ*^2^*p <* 0.05). Those in the Coarse and Detailed groups overwhelmingly reported receiving sufficient information (60% and 54%, respectively) compared with participants in the Absent group, who presented a higher prevalence of somewhat insufficient (31%) and insufficient (19%) information. The coarse group had the smallest proportion of participants who found the FOP to be somewhat insufficient to inform choices in the choice task (22.0%).

**Table 10:** Subjective FOP understanding questions by experimental group (*n* = 498)

Variable	Alternative	All *n* (%)	Absent *n*(%)	Coarse *n* (%)	Detailed *n*(%)	χ2 (*p*)
Participants		498	162 (32)	167 (34)	169 (34)	
Reported seeing FOP	Always	147 (44)		80 (48)	67 (40)	0.22
Reported seeing FOP	Most of the time	104 (31)		51 (36)	53 (31)	
Reported seeing FOP	About half the time	32 (10)		10 (6)	22 (13)	
Reported seeing FOP	Sometimes	39 (11)		19 (11)	20 (12)	
Reported seeing FOP	Never	14 (4)		7 (4)	7 (4)	
Having information	Overload or sufficient	244 (49)	54 (33)	99 (60)	91 (54)	0.00
Having information	Neither	66 (13)	26 (16)	19 (11)	21 (12)	
Having information	Somewhat insufficient	133 (27)	51 (31)	36 (22)	46 (27)	
Having information	Insufficient	55 (11)	31 (19)	13 (8)	11 (7)	

### Robustness checks

The multilevel linear regression analysis on calorie count investigated six specific groups × n-back interactions (see Appendix D, Table 1). Prior to adjusting for multiple comparisons, four of these six interactions reached nominal significance. However, after applying Holm’s familywise correction criterion at *α* = 0.05, none of the variables remained significant. Nevertheless, three interactions involving the detail-label condition retained significance under the less stringent Benjamini–Hochberg procedure. In the multilevel log-binomial models, we examined 16 interaction contrasts (see Appendix D, Table 2). None of the 16 contrasts survived Holm’s correction, and only the Detailed*3-back interaction at 392 kcal remained significant at the 5% false discovery rate (FDR) threshold. This attenuation likely stems from the statistical penalty associated with evaluating numerous contrasts on a limited sample rather than a complete absence of genuine effects. Therefore, the corrected results should be interpreted as conservative estimates, with effect size estimates and their confidence intervals providing a clearer indication of practical significance.

To assess the robustness of our findings to downsampling across n-back blocks, we performed a sensitivity analysis via a matched sample approach. Specifically, we rerun all multilevel regression models using only participants (*N* = 452) who completed the three n back blocks, thus holding the sample size constant across working memory conditions. The results from these models were highly consistent with those from the full-sample analyses (see Appendix A). The direction and magnitude of the n-back coefficients remained stable, indicating that the relationship between working memory and FOP effects is not driven by differential dropout. However, the confidence intervals were wider in the full sample, and only the interaction between the detailed label condition and 3-back performance remained statistically significant in the matched sample. This pattern is expected given the reduced sample size and associated loss of statistical power. Furthermore, the completion rate for the sequence of n-back tasks was notably high and was largely independent of the demographic characteristics of the participants (see Appendix E). Chi-square analyses comparing individuals who completed the entire sequence with those who discontinued after the 1 or 2 back blocks revealed no significant differences across the variables examined. Although ethnicity demonstrated a statistically significant association, the effect size was small and diminished to nonsignificance upon collapsing sparse categories, suggesting that the result was driven by low cell counts rather than a systematic pattern of dropout. Collectively, these results suggest that attrition across the n-back blocks was minimal and effectively random with respect to the demographics measured, thus reducing concerns that bias from selective dropout could influence working memory analyses.

## Discussion

This randomized controlled trial offers preliminary evidence suggesting that the effectiveness of FOP labels is partially dependent on and can be optimized for consumer working memory capacity. Across the choice experiment, enhanced n-back performance was consistently correlated with reduced calorie selection under both coarse and detailed label formats, with the most pronounced decrease observed during the demanding 3-back block. Calorie-count models indicated that each one-unit increase in 3-back *d’* reduced purchase calories by approximately 14 kcal with coarse labels and 18 kcal with detailed labels. The Detailed-versus-Absent contrast at the highest capacity level yielded an effect size of − 0.28, which is small but practically significant when it is extrapolated over multiple shopping instances. Multilevel log-binomial regression analyses further indicated that individuals with greater capacity were less likely to select the lowest-calorie option when viewing coarse labels but were more inclined to choose a moderate-calorie product when viewing detailed labels. While these interactions are context-specific and generally small, they suggest a consistent pattern rather than chance.

These findings partially validate the role of cognitive stratification in label effectiveness. They indicate that consumers may employ different processing strategies on the basis of their working memory capacity and the level of detail provided by the labels, as recent work has validated. For example, a comprehensive post hoc analysis of an international cross-sectional survey with a sample of 3,680 adults across 18 nations revealed parallel findings: less than 50% noticed the detailed nutrient-warning FOP during simulated choices, and of those, only one-third could identify the least-healthy option [51]. This suggests that the diagnostic advantages of detailed warning schemes may diminish when label salience or cognitive resources are limited. Similarly, eye tracking in an augmented-reality grocery setting shows that shoppers devote 20–30% fewer fixations to the MTL labels rather than simple “healthy choice” icons, which we interpret as a consequence of the cognitive demand [34]. Furthermore, a recent meta-analysis focusing on individuals aged 16 to 35 years revealed that granular-indicator systems yield the most consistent reductions in calorie selection, followed by color-coded and positive-logo designs; conversely, purely numerical formats have minimal impacts on healthier purchasing [36]. These collective findings emphasize that detailed labels enhance dietary outcomes only when the target demographic possesses both the opportunity and the cognitive capacity to process the information [34, 35].

A total reduction of 4.56% in the average calorie count was observed when the FOP labels were combined with those of the control group, considering 3-back as a moderator. This variation represents a decrease of 3.88% in the coarse group and 5.24% in the detailed group, where 4–5% represents approximately 100–110 calories of a typical 2.000 calorie diet. This reduction is comparable to skipping a 330 ml sugar-sweetened beverage from daily intake, which typically contains approximately 35–40 g of free sugars. Population modelling studies suggest that reducing daily free sugar intake by 30 g can lead to an average body weight reduction of 1.2–1.6 kg over one year and decrease the incidence of type 2 diabetes by 2–3% [52, 53]. An observational study estimated a similar calorie reduction of 6.5% in calories from purchased cereals after the introduction of compulsory warning labels in Chile [14]. Likewise, research conducted in the United States of America indicated a 3% reduction in the average calorie amount under comparable FOP label conditions during the product selection task [41]. In contrast, the results of this study revealed that Coarse was associated with greater calorie reduction than was the control. The findings of the present study diverge from ours, which can be attributed to variations in measurement approaches.

For moderate-calorie products, the positive and statistically significant interaction terms indicate that participants with higher 3-back *d’* scores were more inclined to choose this mid-range product when either label was present. However, at a slightly elevated caloric level of 398 kcal, the interactions were not significant. Furthermore, for the higher-calorie range, none of the interaction terms reached statistical significance, although the Detailed*3-back coefficients consistently exhibited a negative trend. Collectively, these findings imply that FOP labels exert their most pronounced capacity-dependent effect by guiding individuals with high cognitive capacity toward moderate-calorie products rather than discouraging them from lower-calorie choices altogether [4, 5, 11, 54]. These mixed results may indicate that individuals’ choices are influenced by their preexisting beliefs about the calorie content of products [2]. Consistent with previous studies, labelling may be most effective for moderately caloric products for which consumers hold inaccurate calorie beliefs, and memory capacity could enhance the effectiveness even for more granular labelling formats [14].

Sensitivity checks indicated that our results are statistically conservative. First, even when a correction for multiple comparisons in the calorie count models was employed, not all the interactions maintained significance, highlighting a modest capacity-dependent advantage of the more granular format. Similarly, within the log-binomial choice models, the detailed*3-back term for the 392 kcal option remained significant at the 5% FDR. While these adjustments reduce statistical power when examining numerous contrasts within a limited sample, the direction and magnitude of the unrelated effects still indicate small but practical impacts. Second, the effect sizes and directions remained largely consistent, as did the detailed*3-back interaction, upon rerunning all multilevel regressions in a matched subsample.

Granular FOP labels can also directly impact consumers’ subjective understanding, influencing their perceptions of how well they have comprehended the information from the label. Descriptive analysis revealed that labelling was generally perceived favourably in terms of visibility and provided sufficient information for decision-making. This factor does not appear to have contributed to the comparative effects between coarse and detailed labelling. Notably, 44% of the participants always remembered seeing the FOP label to which they were randomized; recall was highest for the coarse label (48%) and lowest for the detailed label (40%). This is important because how labels are noticed plays a crucial role in engagement, as emphasized in Grunert and Wills’ conceptual framework [19]. Despite generally performing better in the choice task, participants in the detailed group reported having sufficient information to choose at a lower rate than those in the coarse group did. The distinction between objective and subjective understanding, where detailed granularity results in fully objective but partially subjective understanding, can be further explored in future studies, including eye-tracking measurements [55].

Labelling is just one factor among many that the participants considered when selecting cereals. For example, the participants indicated a high degree of familiarity, at a rate of 80%, with all cereal products included in the experiment and, on average, ranked their preferences for these items as 3.59, which reflects a moderately strong preference. This finding indicates a potential link between familiarity, preferences, and the use of nutritional information, which may alleviate the cognitive load associated with processing more comprehensive labels. This finding aligns with previous studies on conjoint experiments, suggesting that FOP labels may influence choices compared with other multiple-product attributes. Previous research has indicated that labelling is one of several factors contributing to parents’ choices of children’s snack products in the conjoint choice setting, alongside visual information and nutritional claims such as high fibre content [56]. However, further investigations are needed to corroborate these results.

These findings enhance the current understanding of FOP labelling policies in three significant ways. First, the data suggest that neither a simplified summary label nor a more granular label provides a universally superior solution. Both label types improved calorie selection, and the hypothesized benefit of reduced granularity was not evident. Second, the evidence indicates that label effectiveness is partially dependent on cognitive resources. Only participants demonstrating higher performance in the 3-back task consistently shifted toward moderate-energy options, suggesting that working-memory capacity influences the ability to utilize detailed label information. Third, multiple-comparison and matched-sample analyses demonstrate that these capacity-dependent effects, while modest in size, are statistically robust and unlikely to result from selective dropout. Collectively, these results suggest that future European Union labelling policies should move beyond a standardized approach [57, 58]. Policymakers could integrate a single interpretive label with supplementary tools—such as digital overlays or layered information formats—allow consumers with lower numeracy skills or limited working-memory capacity to reduce informational complexity while still providing more detailed data to those who can utilize it [59]. Tailoring label granularity to consumers’ cognitive resources could therefore maximize public health benefits without sacrificing informational completeness.

### Limitations

This study has several notable strengths. Our study employed prespecified protocols and analysis plans, which were built upon prior research [17]. We developed a choice task involving a set of cereals rather than individual items, as prior research has mentioned that FOP labels perform more effectively in the context of multiple product options available in a grocery setting. This serves to enhance the external validity of our findings. Additionally, our study focused on examining the isolated effects of label granularity by including no-colour labelling options in the choice task rather than incorporating coloured variants. Despite these advantages, the research design has several limitations. The study incorporated a main effects analysis, employing procedures to control the familywise error rate via the Holm method. This analysis revealed that the contrasts did not reach significance, suggesting that our observed moderated effects should be interpreted cautiously pending further replication.

We acknowledge that the online setting, literate and technology-savvy sample, and single food category limit the generalizability of our findings to real-world shopping environments. For example, online settings offer fewer distractions than do busy in-store environments. Online shoppers can easily search and filter products, an option that may not be available in physical stores, and online shoppers lack the sensory cues that can influence food choices in real life. The experiment did not include back-of-pack information despite its availability in real-world settings where additional data could further inform decision-making. Furthermore, factors such as brand loyalty, pricing, shelf placement, and packaging cues were not considered in our study but could influence consumer behavior in real-world settings. Future research should validate our findings in real-world shopping environments by, for example, conducting experiments in actual grocery stores to observe consumer behavior in a more realistic setting [12].

Other aspects of the study design may have reduced the external validity of the findings. It is possible that the analysis included participants who did not fully complete the working memory test instructions. Excluding these individuals from the sample size did not yield any significant effects; nonetheless, the results should be interpreted as indicative of behavioural trends rather than definitive conclusions. The study employed an n-back test to measure working memory, but alternative approaches could be utilized to relate label granularity effectiveness to cognitive capacity. Finally, despite the researchers’ efforts to design images that facilitated accurate selections across all the items, the possibility cannot be ruled out that the detailed labels were unsuitable and challenging for some users, leading to them being overlooked.

## Conclusions

This study provides evidence that FOP calorie labels can significantly influence the energy content of cereal choices. The magnitude and direction of this influence are contingent on both the granularity of the label and the working-memory capacity of consumers. Compared with the absence of labelling, FOP labels reduced average calorie selection by 4.56%, with the detailed format leading to the most substantial reduction and the coarse format resulting in a smaller decrease. Notably, these overall effects concealed a strong capacity-dependent trend: each one-unit increase in 3-back performance corresponded to an additional 14 kcal reduction with coarse labels and 18 kcal with detailed labels. High-capacity shoppers demonstrated a reduced likelihood of selecting the highest-calorie cereals and an increased preference for moderate-calorie options when presented with detailed labels. Collectively, these results offer partial validation for cognitive stratification theory, suggesting that consumers utilize various decision-making approaches on the basis of their working-memory resources and the informational demands of the packaging. Therefore, adapting label granularity to match consumers’ cognitive resources could optimize public-health outcomes without compromising the comprehensiveness of information.

## Supplementary Information

Below is the link to the electronic supplementary material.

Supplementary Material 1

## Data Availability

The datasets used and/or analysed during the current study are available from the corresponding author upon reasonable request.

## Abbreviations

UK: United Kingdom
FOP: Front-of-package label
HSR: Health Star Rating
WL: Warning Labels
MTL: Multiple Traffic Labels
NS: Nutri-Score
KH: Keyhole
BMI: Body Mass Index
RI: Reference Intake
kcal: Kilocalories
g: Grams
d’: d-prime
SD: Standard Deviation
ANOVA: Analysis of Variance
SE: Standard Error

## References

[1] Afshin A, Sur PJ, Fay KA, Cornaby L, Ferrara G, Salama JS, Mullany EC, Abate KH, Abbafati C, Abebe Z, et al. Health effects of dietary risks in 195 countries, 1990–2017: a analysis for the global burden of disease study 2017. Lancet. 2019;393(10184):1958–72. 30954305 10.1016/S0140-6736(19)30041-8 PMC6899507

[2] Fichera E, Hinke S. The response to nutritional labels: evidence from a quasi experiment. J Health Econ. 2020;72:102326. 32526549 10.1016/j.jhealeco.2020.102326

[3] Ikonen I, Sotgiu F, Aydinli A, Verlegh PWJ. Consumer effects of front-of- package nutrition labelling: an interdisciplinary meta-analysis (2020) 10.1007/s11747-019-00663-9

[4] Shangguan S, Afshin A, Shulkin M, Ma W, Marsden D, Smith J, Saheb- Kashaf M, Shi P, Micha R, Imamura F, Mozaffarian D. A meta-analysis of food labelling effects on consumer diet behaviors and industry practices. Ameri- Can J Prev Med. 2019;56:300–14. 10.1016/J.AMEPRE.2018.09.024. 10.1016/j.amepre.2018.09.024 PMC6340779 30573335

[5] Croker H, Packer J, Russell SJ, Stansfield C, Viner R. Front of pack nutri- Tional labelling schemes: a systematic review and meta-analysis of recent evidence relating to objectively measured consumption and purchasing. J Hum Nutr Dietetics. 2020;33(4):518–37. 10.1111/jhn.12758 32364292

[6] Araya S, Elberg A, Noton C, Schwartz D. Identifying food labelling effects on consumer behavior. SSRN Electron J. 2019. 10.2139/SSRN.3195500.

[7] Griffith R, O’Connell M, Smith K. The importance of product reformulation versus consumer choice in improving diet quality. Economica. 2017;84. 10.1111/ecca.12192.

[8] Hawkes C. Nutrition labels and health claims: the global regulatory environment. In: Nutrition Labels and Health Claims: the Global Regulatory Environment, p. 74 (2004).

[9] Helfer P, Shultz TR. The effects of nutrition labelling on consumer food choice: a psychological experiment and computational model. Ann N Y Acad Sci. 2014;1331(1):174–85. 24913496 10.1111/nyas.12461

[10] Allais O, Etil´e F, Lecocq S. Mandatory labels, taxes and market forces: an empirical evaluation of fat policies. J Health Econ. 2015;43:27–44. 10.1016/J.JHEALECO.2015.06.003. 26164818 10.1016/j.jhealeco.2015.06.003

[11] Taillie LS, Hall MG, Popkin BM, Ng SW, Murukutla N. Experimental studies of front-of-package nutrient warning labels on sugar-sweetened beverages and ultra-processed foods: a scoping review. Nutrients. 2020;12(2):569. 32098363 10.3390/nu12020569 PMC7071470

[12] Finkelstein EA, Ang FJL, Doble B. Randomized trial evaluating the effec- Tiveness of within versus across-category front-of-package lower-calorie labelling on food demand. BMC Public Health. 2020;20:1–10. 32164634 10.1186/s12889-020-8434-1 PMC7068974

[13] Loewenstein G, Sunstein CR, Golman R. Disclosure: psychology changes everything. Annu Rev Econ. 2014;6(1):391–419.

[14] Barahona N, Otero C, Otero S. Equilibrium effects of food labelling policies. Econometrica. 2023;91(3):839–68.

[15] Cartwright E. Behavioral Economics, (2018).

[16] Liu PJ, Wisdom J, Roberto CA, Liu LJ, Ubel PA. Using behavioral eco- nomics to design more effective food policies to address obesity. Appl Economic Perspect Policy. 2014;36:6–24. 10.1093/aepp/ppt027.

[17] Scarborough P, Hodgkins C, Raats MM, Harrington RA, Cowburn G, Dean M, Doherty A, Foster C, Juszczak E, Matthews A, et al. Protocol for a pilot randomized controlled trial of an intervention to increase the use of traffic light food labelling in Uk shoppers (the Flicc trial). Pilot Feasibility Stud. 2015;1:1–11. 27965800 10.1186/s40814-015-0015-1 PMC5153808

[18] Green EC, Murphy EM, Gryboski K. The health belief model. Wiley Encyclopedia Health Psychol, 211–4 (2020).

[19] Grunert KG, Wills JM. A review of European research on consumer response to nutrition information on food labels. J Public Health. 2007;15:385–99.

[20] Talati Z, Egnell M, Hercberg S, Julia C, Pettigrew S. Consumers’ percep- tions of five front-of-package nutrition labels: an experimental study across 12 countries. Nutrients. 2019;11(8):1934. 31426450 10.3390/nu11081934 PMC6723043

[21] Dubois P, Albuquerque P, Allais O, Bonnet C, Bertail P, Combris P, Lahlou S, Rigal N, Ruffieux B, Chandon P. Effects of front-of-pack labels on the nutritional quality of supermarket food purchases: evidence from a large- scale randomized controlled trial. J Acad Mark Sci Volume. 2021. 10.1007/s11747-020-00723-5/Published.

[22] Crosetto P, Lacroix A, Muller L, Ruffieux B. Nutritional and economic impact of five alternative front-of-pack nutritional labels: experimental evidence (2020) 10.1093/erae/jbz037

[23] Egnell M, Ducrot P, Touvier M, All`es B, Hercberg S, Kesse-Guyot E, Julia C. Objective Understanding of nutri-score front-of-package nutrition label according to individual characteristics of subjects: comparisons with other format labels. PLoS ONE. 2018;13(8):0202095. 10.1371/journal.pone.0202095 PMC6107140 30138359

[24] Crosetto P, Muller L, Ruffieux B. Helping consumers with a front-of- pack label: numbers or colors? Experimental comparison between guideline daily amount and traffic light in a diet-building exercise. J Econ Psychol. 2016;55:30–50. 10.1016/J.JOEP.2016.03.006.

[25] Taillie LS, Reyes M, Colchero MA, Popkin B, Corval´an C. An evaluation of chile’s law of food labelling and advertising on sugar-sweetened beverage pur- chases from 2015 to 2017: A before-and-after study. PLoS Med. 2020;17:1003015. 10.1371/journal.pmed.1003015. Times cited: 1 PMID: 32045424 PMCID: PMC7012389. 10.1371/journal.pmed.1003015 PMC7012389 32045424

[26] Mazzu` MF, Romani S, Gambicorti A. Effects on consumers’ subjective under- standing of a new front-of-pack nutritional label: a study on Italian consumers. Int J Food Sci Nutr. 2021;72(3):357–66. 32746654 10.1080/09637486.2020.1796932

[27] Packer J, Russell SJ, Ridout D, Conolly A, Jessop C, Viner RM, Cro- ker H. Secondary outcomes of a front-of-pack-labelling randomized controlled experiment in a representative British sample: understanding, ranking speed and perceptions. Nutrients. 2022;14(11):2188. 35683988 10.3390/nu14112188 PMC9182518

[28] Kahneman D. A perspective on judgment and choice: mapping bounded rationality. Am Psychol. 2003;58:697–720. 10.1037/0003-066X.58.9.697. 14584987 10.1037/0003-066X.58.9.697

[29] Wilson A. Bounded memory and biases in information processing. Econometrica. 2014;82:2257–94. 10.3982/ECTA12188.

[30] Lindig-Le´on C, Kaur N, Braun DA. From bayes-optimal to heuristic decision- making in a two-alternative forced choice task with an information-theoretic bounded rationality model. Front NeuroSci. 2022;16:906198. 36248642 10.3389/fnins.2022.906198 PMC9557085

[31] Donini LM, Berry EM, Folkvord F, Jansen L, Leroy F, S¸im¸sek O¨, Fava ., Gobbetti F, Lenzi M. Front-of-pack labels:directive versus informative approaches. Nutrition. 2023;105:111861. 36401998 10.1016/j.nut.2022.111861

[32] Siegrist M, Leins-Hess R, Keller C. Which front-of-pack nutrition label is the most efficient one? The results of an eye-tracker study. Food Qual Prefer. 2015;39:183–90. 10.1016/j.foodqual.2014.07.010. Times cited: 1.

[33] Schruff-Lim E-M, Van Loo EJ, Kleef E, Trijp HC. Turning Fop nutrition labels into action: A systematic review of label + interventions. Food Policy. 2023;120:102479.

[34] Pini V, Orso V, Pluchino P, Gamberini L. Augmented grocery shopping: fostering healthier food purchases through ar. Virtual Reality. 2023;27(3):2117–28.

[35] Pettigrew S, Jongenelis M, Maganja D, Hercberg S, Julia C. The abil- Ity of nutrition warning labels to improve Understanding and choice outcomes among consumers demonstrating preferences for unhealthy foods. J Acad Nutr Dietetics. 2024;124(1):58–64. 10.1016/j.jand.2023.08.135 37673335

[36] Guo Z, Ning Y, Mustafa M. Impact of five types of front-of-package nutrition labels on consumer behavior among young adults: A systematic review. Nutrients. 2024;16(17):2819. 39275139 10.3390/nu16172819 PMC11397554

[37] Bapat SS, Patel HK, Sansgiry SS. Role of information anxiety and informa- Tion load on processing of prescription drug information leaflets. Pharmacy. 2017;5(4):57. 29035337 10.3390/pharmacy5040057 PMC5748538

[38] Ma WJ, Husain M, Bays PM. Changing concepts of working memory. Nat Neurosci. 2014;17(3):347–56. 24569831 10.1038/nn.3655 PMC4159388

[39] Dell’Acqua R, Sessa P, Brigadoi S, Gervain J, Luria R, Doro M. On the functional independence of numerical acuity and visual working memory. Front Psychol. 2024;15:1335857. 38544511 10.3389/fpsyg.2024.1335857 PMC10965769

[40] Lewis NA Jr, Earl A. Seeing more and eating less: effects of portion size granularity on the perception and regulation of food consumption. J Personal Soc Psychol. 2018;114(5):786. 10.1037/pspp0000183 29672105

[41] Ravaioli S. Coarse and precise information in food labelling. Job Market Paper, 1 (2021). Times cited: 1.

[42] Agency FS. Guide to creating a front of pack (FoP) nutrition label for pre- packed products sold through retail outlets. UK: Department of Health of UK London; 2016.

[43] Holleman GA, Hooge IT, Kemner C, Hessels RS. The ‘real-world approach’and its problems: A critique of the term ecological validity. Front Psychol. 2020;11:721. 32425850 10.3389/fpsyg.2020.00721 PMC7204431

[44] Bradley-Garcia M, Bolton V. Programming an n-back task in qualtrics using Html and javascript. Univ Ott 19 (2023).

[45] Golmohammadi R, Darvishi E, Faradmal J, Poorolajal J, Aliabadi M. Attention and short-term memory during occupational noise exposure considering task difficulty. Appl Acoust. 2020;158:107065.

[46] Schmidt H, Jogia J, Fast K, Christodoulou T, Haldane M, Kumari V, Frangou S. No gender differences in brain activation during the n-back task: an Fmri study in healthy individuals. Hum Brain Mapp. 2009;30(11):3609–15. 19387979 10.1002/hbm.20783 PMC6870785

[47] Jaeggi SM, Buschkuehl M, Perrig WJ, Meier B. The concurrent validity of the n-back task as a working memory measure. Memory. 2010;18(4):394–412. 20408039 10.1080/09658211003702171

[48] Jun E, Hsieh G, Reinecke K. Types of motivation affect study selection, attention, and dropouts in online experiments. Proc ACM Hum Comput Interact. 2017;1(CSCW):1–15.

[49] R Core Team: R: A Language and Environment for Statistical Computing. R Foundation for Statistical Computing, Vienna, Austria. (2021). R Foundation for Statistical Computing. https://www.Rproject.org/

[50] Stanislaw H, Todorov N. Calculation of signal detection theory measures. Behavior research methods. Instruments Computers. 1999;31(1):137–49. 10.3758/bf03207704 10495845

[51] Pettigrew S, Dana LM, Talati Z, Tian M, Praveen D. The role of colour and summary indicators in influencing front-of-pack food label effectiveness across seven countries. Public Health Nutr. 2021;24(11):3566–70. 33317658 10.1017/S1368980020004966 PMC10195333

[52] Imamura F, O’Connor L, Ye Z, Mursu J, Hayashino Y, Bhupathiraju SN, Forouhi NG. Consumption of sugar sweetened beverages, artificially sweet- Ened beverages, and fruit juice and incidence of type 2 diabetes: systematic review, meta-analysis, and Estimation of population attributable fraction. BMJ 351 (2015). 10.1136/bmj.h3576 PMC4510779 26199070

[53] Scientific Advisory Committee on Nutrition: Carbohydrates and health. Technical report, England PH. London (2015). ISBN 708284-7978-0-11. https://assets.publishing.service.gov.uk/government/uploads/system/uploads/attachmentdata/file/445503/SACN Carbohydrates and Health.pdf.

[54] Crockett RA, King SE, Marteau TM, Prevost AT, Bignardi G, Roberts NW, Stubbs B, Hollands GJ, Jebb SA. Nutritional labelling for healthier food or nonalcoholic drink purchasing and consumption. The Cochrane database of systematic reviews 2018(2) (2018). 10.1002/14651858.CD009315.pub2 PMC5846184 29482264

[55] Mazzu` MF, Baccelloni A, Finistauri P. Uncovering the effect of European policy-making initiatives in addressing nutrition-related issues: a systematic liter- ature review and bibliometric analysis on front-of-pack labels. Nutrients. 2022;14(16):3423. 36014929 10.3390/nu14163423 PMC9414449

[56] Velazquez AL, Alcaire F, Vidal L, Varela P, Næs T, Ares G. The influence of label information on the snacks parents choose for their children: individual differences in a choice based conjoint test. Food Qual Prefer. 2021;94:104296.

[57] Delhomme V. Front-of-pack nutrition labelling in the European union: a behavioural, legal and political analysis (2021) 10.1017/err.2021. 5.

[58] Kelly B, Jewell J. What is the evidence on the policy specifications, Devel- opment processes and effectiveness of existing Front-of-pack food labelling policies in the WHO European region?? World Health Organization. Regional Office for Europe; 2018. 30484994

[59] Zarantonello L, Schiff S, Amodio P, Bisiacchi P. The effect of age, educational level, gender and cognitive reserve on visuospatial working memory performance across adult life span. Aging Neuropsychol Cognition. 2020;27(2):302–19. 10.1080/13825585.2019.1608900 31046560

