# Supplementary material for: Food label granularity and working memory: effects on food choice in a randomized controlled trial
Source: J Health Popul Nutr. 2025 Oct 24;44:375. doi: 10.1186/s41043-025-01076-x (PMC12553146; doi:10.1186/s41043-025-01076-x)
Supplement: Supplementary file 1 — Supplementary Material 1 [file 41043_2025_1076_MOESM1_ESM.pdf]

Appendix A

	Model 1	Model 2	Model 3	Model 4	Model 5	Model 6
(Intercept)	375.62***	392.48***	357.19***	353.81***	350.42***	361.93***
	(5.18)	(5.55)	(43.38)	(46.48)	(47.09)	(44.64)
Coarse	-10.86	-10.86	-5.29	30.30	29.95	26.06
	(7.34)	(7.34)	(6.76)	(29.68)	(27.06)	(19.13)
Detailed	-8.97	-8.97	-8.70	14.40	34.40	30.66*
	(7.27)	(7.27)	(6.65)	(28.46)	(26.87)	(17.74)
1-back				0.09		
				(5.67)		
Coarse*1-back				-10.17		
				(8.26)		
Detailed*1-back				-6.68		
				(7.96)		
2-back					2.63	
					(5.80)	
Coarse*2-back					-10.63	
					(7.90)	
Detailed*2-back					-13.30	
					(7.93)	
3-back						1.03
						(5.38)
Coarse*3-back						-13.52
						(7.83)
Detailed*3-back						-18.05**
						(7.42)
AIC	87016.62	86949.75	86846.64	86832.41	86830.63	86821.42
Num. obs.	7232	7232	7232	7232	7232	7232
Num. groups: Response ID	452	452	452	452	452	452

$^{***}p < 0.01$; $^{**}p < 0.05$; $^{*}p < 0.1$

Table 1: Multilevel linear regression results: effects of FOP labelling on calorie count by experimental conditions, including n-back test performance levels as an interaction term (adjusted for product preferences and choice sets (Trials)

Appendix B

	0	360	374	392	398	423	431	453	470
(Intercept)	-9.04***	-4.35***	7.11***	-2.54***	-6.85***	-4.00***	-2.50***	-6.64***	-8.25***
	(3.07)	(0.81)	(0.53)	(0.91)	(1.35)	(0.76)	(0.85)	(1.51)	(1.58)
Coarse	-2.57	0.97	-0.38	0.30	0.29	1.22	0.11	0.02	-0.70
	(1.60)	(1.08)	(0.71)	(1.23)	(1.75)	(1.03)	(1.19)	(1.94)	(2.21)
Detailed	-2.15	-0.63	-0.03	1.32	1.50	0.24	0.29	1.82	0.55
	(1.54)	(1.05)	(0.72)	(1.17)	(1.69)	(1.01)	(1.14)	(1.85)	(2.09)
2-back	-0.20	-0.08	-0.18	0.03	-0.06	0.55***	0.12	0.06	-0.07
	(0.31)	(0.23)	(0.14)	(0.25)	(0.34)	(0.20)	(0.23)	(0.38)	(0.42)
Coarse*2-back	0.81*	-0.30	0.18	0.26	0.17	-0.57*	-0.48	-0.03	0.06
	(0.47)	(0.33)	(0.21)	(0.36)	(0.52)	(0.30)	(0.35)	(0.57)	(0.66)
Detailed*2-back	0.74	0.23	0.04	-0.06	-0.25	-0.24	-0.44	-0.48	-0.30
	(0.46)	(0.32)	(0.22)	(0.35)	(0.51)	(0.30)	(0.34)	(0.56)	(0.64)
AIC	3000.03	2597.38	3118.16	4163.87	3009.89	4494.93	3779.43	2401.06	2313.40
Num. obs.	7424	7424	7424	7424	7424	7424	7424	7424	7424
Num. groups:	464	464	464	464	464	464	464	464	464

***$p < 0.01$; **$p < 0.05$; *$p < 0.1$

Table 1: Multilevel log-binomial regression results—effects of FOP labelling on the probability of choosing lower-calorie cereal brands by experimental groups with the 2-back level as the interaction term (adjusted for ranking preferences and trials)

	0	360	374	392	398	423	431	453	470
(Intercept)	-8.93***	-4.09***	6.08***	-2.78***	-7.39***	-3.03***	-2.68***	-7.46***	-8.39***
	(3.10)	(0.79)	(0.53)	(0.88)	(1.37)	(0.78)	(0.83)	(1.54)	(1.60)
Coarse	-0.45	-0.38	0.12	2.55***	0.81	-0.62	-0.68	0.45	-0.26
	(1.41)	(0.91)	(0.60)	(0.99)	(1.54)	(0.89)	(1.02)	(1.70)	(1.80)
Detailed	-1.84	-0.01	0.13	1.72*	0.82	0.09	-0.44	0.92	-0.02
	(1.51)	(0.92)	(0.63)	(1.02)	(1.57)	(0.89)	(1.03)	(1.73)	(1.85)
1-back	0.01	-0.15	0.06	0.07	-0.03	0.23	0.14	0.21	-0.03
	(0.32)	(0.21)	(0.14)	(0.23)	(0.34)	(0.20)	(0.22)	(0.38)	(0.41)
Coarse*1-back	0.17	0.09	0.02	-0.39	0.02	-0.01	-0.26	-0.16	-0.09
	(0.40)	(0.27)	(0.18)	(0.28)	(0.44)	(0.25)	(0.29)	(0.49)	(0.52)
Detailed*1-back	0.59	0.02	-0.01	-0.15	-0.05	-0.18	-0.18	-0.17	-0.10
	(0.43)	(0.27)	(0.18)	(0.29)	(0.45)	(0.26)	(0.30)	(0.50)	(0.53)
AIC	3192.82	2784.73	3477.17	4640.75	3234.31	4747.26	3978.20	2518.29	2457.71
Num. obs.	7968	7968	7968	7968	7968	7968	7968	7968	7968
Num. groups:	498	498	498	498	498	498	498	498	498

$^{***}p < 0.01$; $^{**}p < 0.05$; $^{*}p < 0.1$

Table 2: Multilevel log-binomial regression results—effects of FOP labelling on the probability of choosing lower-calorie cereal brands by experimental groups with the 1-back level as the interaction term (adjusted for ranking preferences and trials)

Appendix D

Model	Interaction	t	p-values	p-Holm	p-BH
Model 4	Coarse*1-back	-1.731	0.0835	0.1669	0.0840
Model 4	Detailed*1-back	-2.465	0.0137	0.0685	0.0299
Model 5	Coarse*2-back	-2.103	0.0355	0.1064	0.0532
Model 5	Detailed*2-back	-2.569	0.0102	0.0612	0.0299
Model 6	Coarse*3-back	-1.728	0.0840	0.1669	0.0840
Model 6	Detailed*3-back	-2.433	0.0150	0.0685	0.0299

Table 1 Interaction t-values with Holm and Benjamini–Hochberg corrections

Model	Interaction	p-values	p-Holm	p-BH
360	Coarse*3-back	0.0256	0.3844	0.2050
360	Detailed*3-back	0.7343	1.0000	0.9968
374	Coarse*3-back	0.3422	1.0000	0.7823
374	Detailed*3-back	0.6228	1.0000	0.9968
392	Coarse*3-back	0.0914	1.0000	0.4874
392	Detailed*3-back	0.0208	0.3326	0.2050
398	Coarse*3-back	0.9903	1.0000	0.9968
398	Detailed*3-back	0.9158	1.0000	0.9968
423	Coarse*3-back	0.4578	1.0000	0.9156
423	Detailed*3-back	0.1351	1.0000	0.5403
431	Coarse*3-back	0.2771	1.0000	0.7390
431	Detailed*3-back	0.1760	1.0000	0.5632
453	Coarse*3-back	0.8312	1.0000	0.9968
453	Detailed*3-back	0.9346	1.0000	0.9968
470	Coarse*3-back	0.9968	1.0000	0.9968
470	Detailed*3-back	0.8609	1.0000	0.9968

Table 2 3-back interaction p-values with Holm and Benjamini–Hochberg corrections

Appendix E

	Block	Completion (\%)
Completed	1-back	100.0
Completed	2-back	93.2
Completed	3-back	90.8

Table 3 N-back block completion rates

Variable	χ^2	df	p	Cramér's V
Sex	1.48	2	0.477	0.055
Age	6.79	5	0.236	0.117
Education level	5.50	5	0.358	0.105
Income	0.93	5	0.968	0.043
Ethnicity	18.85	5	0.002	0.195
Children at home	1.31	2	0.520	0.051
Shopping responsibility	7.14	4	0.129	0.120
Trying to loose weight	4.07	2	0.131	0.090
BMI	394.63	410	0.699	0.890

Table 4 Chi-square comparison of full vs. partial completers on demographics

Appendix F

1.1 Sample

We conducted an a priori power analysis via G*Power 3.1 to determine the sample size of the experiment. The analysis aimed for 85% power (β = 0.15) to detect a medium-sized interaction effect (Cohen's h = 0.15, two-tailed = 0.05). This produced a minimum required sample size of 163 participants per condition (3 $\times$ 163 = 489 in total). To account for potential attrition or incomplete responses that may require exclusion, we set a final sample size approximately 10% larger than the calculated minimum.

1.2 Trial randomization

Products were classified into calorie bins labelled Very low, Low, High, and Very high to obtain combinations that included at least one cereal from each of these bins while excluding any sets containing cereals from the same classification. Additionally, randomization also included the position of the four cereals within each trial and trial order to mitigate any potential positional biases. Each cereal product was presented an equal number of times. This design ensured that each trial presented participants with a diverse range of calorie options while preventing redundancy within calorie levels.

To minimize missing data, participants can select the option *I would not choose any of them* after attempting to move on to the next phase without completing the choice task. Qualtrics prevent the selection of two products at the same time. The participants could zoom in on the image but were not provided with any other information regarding calories. The subjects selected cereals at their own pace, while the survey software recorded the length of time spent (in seconds). We advised participants to click 'Next' as soon as they had completed each task.
